# Group B streptococcal carriage, serotype distribution and antibiotic susceptibilities in pregnant women at the time of delivery in a refugee population on the Thai-Myanmar border

**DOI:** 10.1186/1471-2334-12-34

**Published:** 2012-02-08

**Authors:** Claudia Turner, Paul Turner, Linda Po, Naw Maner, Aruni De Zoysa, Baharak Afshar, Androulla Efstratiou, Paul T Heath, François Nosten

**Affiliations:** 1 Shoklo Malaria Research Unit, Mae Sot 63110, Thailand; 2 Mahidol-Oxford Tropical Medicine Research Unit, Bangkok 10400, Thailand; 3 Centre for Tropical Medicine, University of Oxford, Oxford OX3 7LJ, UK; 4 Microbiology Services Division, Colindale, Health Protection Agency, 61 Colindale Ave, London NW9 5EQ, UK; 5 St George's, University of London, Cranmer Terrace, London SW17 0RE, UK

## Abstract

**Background:**

Group B Streptococcus (GBS) is the leading cause of neonatal sepsis in the developed world. Little is known about its epidemiology in the developing world, where the majority of deaths from neonatal infections occur. Maternal carriage of GBS is a prerequisite for the development of early onset GBS neonatal sepsis but there is a paucity of carriage data published from the developing world, in particular South East Asia.

**Methods:**

We undertook a cross sectional study over a 13 month period in a remote South East Asian setting on the Thai-Myanmar border. During labour, 549 mothers had a combined vaginal rectal swab taken for GBS culture. All swabs underwent both conventional culture as well as PCR for GBS detection. Cultured GBS isolates were serotyped by latex agglutination, those that were negative or had a weak positive reaction and those that were PCR positive but culture negative were additionally tested using multiplex PCR based on the detection of GBS capsular polysaccharide genes.

**Results:**

The GBS carriage rate was 12.0% (95% CI: 9.4-15.0), with 8.6% positive by both culture and PCR and an additional 3.5% positive by PCR alone. Serotypes, Ia, Ib, II, III, IV, V, VI and VII were identified, with II the predominant serotype. All GBS isolates were susceptible to penicillin, ceftriaxone and vancomycin and 43/47 (91.5%) were susceptible to erythromycin and clindamycin.

**Conclusions:**

GBS carriage is not uncommon in pregnant women living on the Thai-Myanmar border with a large range of serotypes represented.

## Background

Each year four million neonates die from neonatal sepsis, the majority in the first week of life in the developing world [1]. Group B streptococcus (GBS, *Streptococcus agalactiae*) is a leading cause of neonatal sepsis in the developed world [2,3]. Historically, infection with GBS has been reported to be rare in the developing world; however its importance as a cause of neonatal sepsis in these countries is being increasingly recognised [4-9].

An estimated 20-30% of women are colonized by GBS in developed countries; however there is a paucity of data regarding GBS colonization in the developing world and in particular South East Asia [10-12]. This is an important gap in knowledge. Not only is there a large geographical variation in both carriage rates and serotype distributions, but also GBS carriage varies temporally [13]. Maternal carriage of GBS is necessary for early neonatal infection to occur, with the gastrointestinal tract being the primary site of colonization [14]. Transmission is thought to occur just before or during birth when GBS ascends the genital tract into the amniotic fluid where it is aspirated or ingested by the infant [12]. Intrapartum antibiotics given to women colonized with GBS have been shown to reduce the incidence of early onset GBS neonatal sepsis [15]. Various strategies for the use of intrapartum antibiotics (IPA) are used globally, with risk-based IPA administration in the UK and a universal culture based screening program in the USA [16,17]. The introduction of intrapartum antibiotics in the developing world could reduce the number of infants who die from neonatal sepsis each year. However to determine the likely impact and the optimal strategy of antibiotic administration the colonization prevalence and neonatal infection rate for GBS in the target population should be known. Another potential strategy for GBS prevention is the introduction of a GBS vaccine. Current candidates are polysaccharide protein conjugates so knowledge of the serotype distribution of GBS in different geographical areas will be important in planning their implementation.

The aim of this study was to describe the GBS colonization rate, serotype distribution and antibiotic sensitivity profile in a rural population in South East Asia.

## Methods

Maela Camp for displaced persons is a densely populated camp predominantly inhabited by Karen refugees from Myanmar (Burma). It is located in North West Thailand in the hills adjoining the Myanmar border 60 km north of Mae Sot. It is the largest of the camps for displaced people on the Thai-Myanmar border, housing around one-third of the total refugee population. Maela has a population of approximately 43,000 people, living in an area of 4 km^2^. The Shoklo Malaria Research Unit (SMRU) clinic provides all antenatal care in the camp, where 1500 deliveries occur each year.

We conducted a cross sectional GBS carriage study at the SMRU clinic between April 2009 and May 2010. All women who attended the antenatal clinic and were between 28 and 30 weeks gestation were asked to participate. Women were asked to deliver at the SMRU clinic so that a vaginal-rectal swab, a venous blood sample and umbilical cord blood could be obtained during labour. It is not possible to carry out Caesarean sections in the camp so all infants are born vaginally. A standard proforma was completed after delivery to determine ethnic group, past obstetric history, recent antibiotic exposure, and complications occurring during labour, concentrating on the risk factors for neonatal GBS infection (fever > 38C, prolonged rupture of membranes, prematurity (< 37 weeks) that are included in risk based strategy for the prevention of GBS neonatal sepsis [16]. All infants were reviewed at planned study visits at seven and 28 days and at unplanned sick visits, to determine the incidence of neonatal mortality and neonatal sepsis.

### Detection of GBS carriage by culture

Combined vaginal-rectal swabs were collected and processed following the CDC guideline [17]. Briefly, a combined vaginal-rectal swab was collected using a sterile swab with Amie's transport medium (Transwab, Medical Wire & Equipment, Corsham, UK). These swabs were refrigerated until culture on the day of collection. The swab tip was cultured overnight at 36°C in 5% CO_2 _in 5 ml LIM broth (Todd-Hewitt broth + colistin and nalidixic acid; prepared in-house). A 5 μL loop of this broth was subsequently sub-cultured on to 5% sheep blood agar (Clinical Diagnostics, Bangkok, Thailand) and incubated for 24-48 hours at 36°C in 5% CO_2_. Morphologically suspected GBS colonies were confirmed by Gram's stain, catalase reaction, and Lancefield grouping by latex agglutination (Streptococcus Grouping kit, Oxoid, Basingstoke, UK). Antimicrobial susceptibilities were determined by disc diffusion following 2009 CLSI methodology [18]. All GBS isolates were serotyped by latex agglutination (Strep-B-Latex kit, Statens Serum Institute, Copenhagen Denmark). Those isolates that were non-typeable by this serotyping method or had a weak positive reaction were sent to the Health Protection Agency Microbiology Services Division Colindale, Streptococcus and Diphtheria Reference Unit for further characterisation using multiplex PCR assays based on the detection of GBS capsular genotypes (Ia, Ib-IX) [19].

### Detection of GBS carriage by polymerase chain reaction (PCR)

A 1 mL aliquot of the cultured LIM broth was stored at -80°C for subsequent Group B Streptococcal PCR. DNA was extracted from 200 μL of the broth specimens using the Bacterial DNA kit for the MagCore HF16 automated nucleic acid extractor (RBC Bioscience, Taipei, Taiwan). The real-time *cfb *gene PCR protocol developed by Ke et al. was modified for use with a Rotorgene 6000 instrument (Corbett Life Science, Australia) [20]. Briefly, the PCR primers (Sag59 and Sag190) were identical to those used in the original method, but a novel Taqman probe (sequence: 5'-CATGCTGATCAAGTGACAACTCCACA-3') was developed. This modified assay was demonstrated to be GBS-specific and to have a limit of detection of < 1 GBS cfu/reaction (unpublished data). All PCRs were run with a negative and positive control (clinical GBS isolate); specimen cT values of < 40, with appropriate run controls, were considered positive. Broth DNA extraction was repeated and PCR rerun in all specimens with a first cT value of 35-39, and only those with repeated low positive cT values and an appropriate size band on agarose gel electrophoresis were considered positive.

With the exception of PCR capsular genotyping, all laboratory work was performed at the SMRU microbiology laboratory in Mae Sot, Thailand: this laboratory submits to the Thailand national microbiology DMSc NEQAS scheme (unit code MI 1158).

### Statistical analysis

Data were entered into an Access 2003 database (Microsoft, Redmond WA, USA) and all statistical analyses carried out using STATA 10.1 (StataCorp, College Station TX, USA). The Student's *t*-test was used to compare means and the two-sample Wilcoxon rank sum to compare medians. The Chi-squared test was used to compare proportions, with two-tailed *p*-values of < 0.05 indicating significance.

### Study ethics

All women gave informed consent to participate in the study. Ethical approval was granted by the Ethics Committee of The Faculty of Tropical Medicine, Mahidol University, Thailand (MUTM 2009-011-03) and the Oxford Tropical Research Ethics Committee, Oxford University, UK (48 08).

## Results

Six hundred and seventy five women were enrolled over the 13 month study period. During labour, 549 women had vaginal-rectal swabs taken; 126 swabs were not obtained as 14 women (2%) were lost during pregnancy and 112 (17%) delivered outside of the SMRU clinic. Epidemiological data for the women who had swabs taken are shown in Table 1; characteristics did not differ between those women who had a swab and those women who did not (data not shown). There were a total of 553 live births and three still births. Of the 549 swabs obtained 47/549 (8.6% [95% CI: 6.4-11.2]) were found to be positive for GBS by culture with an additional 19 samples positive by PCR, making the overall GBS carriage rate 66/549 (12.0% [95% CI: 9.4-15.0]). Comparing culture to PCR, the sensitivity of culture was 71% (Table 2). There were no swabs that were GBS culture positive but PCR negative.

**Table 1:** Ethnicity, previous number of pregnancies, miscarriage and neonatal deaths for all women who had a vaginal-rectal swab taken (n = 549)

Epidemiological information	Number (%)
Maternal Ethnic Group	

Karen	442 (80.3)

Muslim	78 (14.3)

Burmese	9 (1.7)

Other	15(2.8)

Missing	5 (0.9)

Gravida	

1	184 (33.5)

2	117 (21.3)

3	92 (16.8)

≥ 4	156 (28.5)

Previous Miscarriage	

0	429 (78.1)

1	90 (16.4)

2	18 (3.3)

≥ 3	9 (1.6)

Missing	3 (0.6)

Previous Neonatal Death	14 (2.6)

**Table 2:** Comparison of PCR versus culture for GBS detection

	GBS PCR negative	GBS PCR positive	Total
**GBS culture negative**	483	19	502

**GBS culture positive**	0	47	47

**Total**	483	66	549

None of the risk factors that form the basis of a GBS risk based IPA strategy (fever, prolonged rupture of membranes, prematurity) were shown to be significantly associated with GBS carrier status (≥ 1 factor in 17% carriers vs. 18% in non-carriers (*p *= 0.7), Table 3) [16]. None of the mothers were known to have previously had a baby with GBS disease. Overall therefore, 100/549 (18.2%) women had one or more clinical risk factors. During labour 44/549 (8%) women received antibiotics: ampicillin was the most commonly prescribed (95%). Only one woman received erythromycin and none received clindamycin. 39/44 (89%) women who received antibiotics did so because of PROM; 2/44 (5%) for a urinary tract infection; 1/44 (2%) for chorioamnionitis and 2/44 (5%) for PROM and a urinary tract infection. 4/47 (8.5%) of the GBS culture positive mothers received antibiotics, all of these were given for PROM. None of the mothers who were GBS culture negative but PCR positive received antibiotics during labour.

**Table 3:** Risk factors for GBS carriage

Risk Factor	GBS carrier (n = 66)	GBS negative (n = 483)	P value
		
	% (95% CI)	% (95% CI)	
Any risk factor	11	89	0.7
	16.7 (8.6-27.9)	18.4 (15.1-22.2)	

Fever > 38°C in labour	0	3	0.5
	0.0 (0.0-5.4)	0.6 (0.1-1.8)	

PROM > 18 hours	4	36	0.6
	6.0 (1.7-14.8)	7.5 (5.3-10.2)	

Preterm < 37 weeks	7	49	0.1
	10.6 (4.4-20.6)	7.4 (5.3-10.2)	

Fever and PROM	0	1	0.7
	0.0 (0.0-5.4)	0.2 (0.0-1.1)	

Fever and preterm	0	2	0.6
	0.0 (0.0-5.4)	0.4 (0.0-1.5)	

PROM and preterm	2	8	0.4
	3.0 (0.4-10.0)	16.6 (0.1-3.2)	

Preterm, PROM & fever	0	1	0.7
	0.0 (0.0-5.4)	0.2 (0.0-1.1)	

*PROM *prolonged rupture of membranes

**Figure 1:**
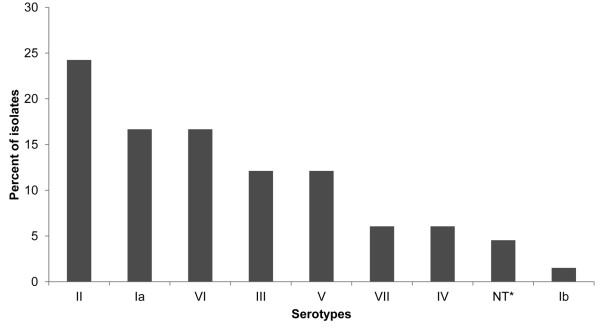
Distribution of GBS capsular serotypes among 549 pregnant women at the time of delivery (*non-typeable).

Eight of the ten currently known GBS serotypes were identified, the most common being serotype II (16/66 isolates); the serotype distribution and frequency is shown in Figure 1. Of those that were culture positive 25/47 (53.2%) of serotypes were identified by latex agglutination alone, with serotype III being the commonest detected. 4/47 (8.5%) were non typeable by latex agglutination and subsequently were genotyped by PCR, with all being identified as capsular genotype VI. Overall 18/47 (38.3%) showed a weak or mixed reaction with latex agglutination but showed a clear capsular genotype with PCR. This was particularly the case with capsular genotype VI where none were clearly identified by latex agglutination. Of the swabs that were culture negative but PCR positive serotypes II and VI were the most frequent (5/19, 26.3% each).

All GBS isolates were susceptible to penicillin, ceftriaxone and vancomycin and 43/47 (91.5%) were susceptable to erythromycin and clindamycin.

There was no increased risk of neonatal sepsis or of neonatal death in infants born to women who were colonized with GBS compared with those who were not colonized. Of the 553 live born infants, 24 developed clinical signs of early neonatal sepsis. Two of these mothers were carriers of GBS (one serotype II and one serotype III) but neither infant grew GBS from either blood or CSF culture. No infant developed late onset GBS sepsis, in fact there were no blood or CSF culture proven episodes of sepsis. There were no early neonatal deaths in infants born to mothers colonized with GBS and only one late neonatal death at 18 days of age, from an accident.

## Discussion

This is the first GBS carriage study to be performed in a rural South East Asian population and it adds to the scant knowledge of GBS carriage in this region and the developing world generally.

The GBS carriage rate in the pregnant women living in Maela camp for displaced persons is 12%. Two previous studies carried out in Thailand showed GBS carriage rates of 16% and 18% [21,22]. The difference in these rates is not unexpected as the study participants were from different ethnic groups and geographic locations, with our study population being more reflective of a developing world country such as Myanmar.

The rate of culture positive GBS carriage was found to be lower than that detected by PCR. The PCR method used in this study has been compared to standard culture techniques and other PCR methods by Rallu et al. When compared to the *scp*B PCR, the *cfb *PCR was found to have a sensitivity of 75.3% and culture a sensitivity of 42.3% [23]. Various hypotheses for this effect have been suggested: antibiotics in the lead up to the time of the swab or suppression of growth of GBS by enterococci present in vaginal and rectal flora. In addition, there may be difficulty in identifying GBS on culture due to overgrowth of Gram positive bacteria from the recto-vaginal swab or due to a light growth of GBS [23,24].

Antibiotic susceptibility remains high in this population with most isolates retaining susceptibility to the macrolides despite reports of increasing erythromycin and clindamycin resistance in GBS carriage isolates in other regions [25]. This most probably reflects the low use of these antibiotics in this population.

A large range of carried serotypes were identified, with only a small number (4.5%) being non typeable. All of the non typeable serotypes were on isolates identified by PCR not on culture positive isolates, which raises the possibility of false positive GBS *cfb *PCR results. However, the cT values for these specimens were well within the expected range. Four GBS culture positive isolates that were initially non typeable by latex agglutination were subsequently identified to be capsular genotype VI by PCR.

A recently published meta-analysis of geographical GBS serotype distribution of carriage isolates, showed that in Canada, North America and South America serotype Ia was the predominant serotype. In Europe, the Middle East, Africa, Australia and Asia serotype III predominated. Serotype II, which was the most common serotype found in this study, was not found to be a predominate serotype in any region [26].

There is very little published data on GBS carriage in Asia and in particular on the serotype distribution, however studies from Japan and Korea have described the GBS serotype distributions in their populations [27,28]. In Korea the predominant serotype was serotype III (20.3%), followed by serotype Ia (12.1%). In the Japanese study serotype VIII was the most common serotype making up 35.6% of isolates. Another common serotype isolated in this study was serotype VI, which is rarely reported in carriage studies from other parts of the world. Interestingly this was the second most common isolate in our study. The frequency of isolation of this serotype was not significantly different to that in our study (24.6% vs 16.7% *p *= 0.3).

One of the strengths of the current study is that GBS serotyping was performed on both culture isolates and PCR positive isolates giving an accurate picture of serotype distribution in this population.

An ongoing study in the same population will define the incidence of early onset neonatal sepsis and the specific role of GBS, with the aim to combine the results of both studies in order to formulate a preventative strategy for GBS neonatal sepsis.

## Conclusions

GBS carriage is not uncommon in pregnant women living on the Thai-Myanmar border with a large range of serotypes represented. These data are important for future global GBS vaccine development and implementation.

## Competing interests

The authors declare that they have no competing interests.

## Authors' contributions

CT, PT, PH and FN conceived the study. NM and CT were responsible for specimen and data collection. LP, AE, BA and PT performed the laboratory work. CT did the data analysis and prepared the first draft of the manuscript. All authors reviewed and contributed to revisions of the manuscript.

## Pre-publication history

The pre-publication history for this paper can be accessed here:

http://www.biomedcentral.com/1471-2334/12/34/prepub
